# Unilateral Renal Artery Vasculitis: A Case Report

**DOI:** 10.31138/mjr.230126.ral

**Published:** 2026-06-12

**Authors:** Iraklis Lefas, Pinelopi Konstantopoulou

**Affiliations:** Rheumatology Department, General Hospital of Athens ‘’G. Gennimatas’’, Athens, Greece

**Keywords:** vasculitis, arteritis, renal artery inflammation

## Abstract

Renal artery inflammation is typically observed as part of systemic vasculitis rather than as an isolated condition. Large vessel vasculitides, such as Takayasu arteritis and giant cell arteritis, as well as polyarteritis nodosa, may involve multiple vessels, including the renal artery. Isolated vasculitis of the renal artery is exceedingly rare with only eight cases reported worldwide. We describe a case of a previously healthy 53-year-old man who presented with a history of right flank pain which was attributed to renal artery vasculitis. Extensive radiologic studies revealed no evidence of inflammation in other vessels and laboratory evaluation for an underlying infectious, neoplastic or autoimmune cause was negative. Treatment with methylprednisolone, followed by azathioprine as a steroid-sparing agent, led to complete remission of the condition.

## INTRODUCTION

Vasculitis is a heterogeneous group of inflammatory disorders that affect the vessel wall, potentially leading to ischemia and end-organ damage.^1^ According to the 2012 Revised International Chapel Hill Consensus Conference, vasculitic syndromes are classified based on the size and distribution of vessel involvement, including large, medium, and small vessel vasculitides, with substantial overlap between these categories.^2^ T h e renal artery, a medium-sized vessel, can be affected in various vasculitides as part of systemic involvement. Large vessel vasculitides, which primarily affect the aorta and its major branches, may involve the renal artery up to 68% of patients with Takayasu arteritis^3^ and 7.5% of those with giant cell arteritis.^4^ Medium vessel vasculitis, such as polyarteritis nodosa, has also been reported to involve the renal and intrarenal arteries in approximately two-thirds of patients.^5^

All forms of vasculitis typically involve multiple vessels simultaneously, as commonly demonstrated by clinical or radiologic evaluations. Isolated involvement of a single vessel is rare and may pose diagnostic and therapeutic challenges.^6^ Here, we present a case of isolated renal artery vasculitis, which manifested as acute right flank pain and microscopic haematuria. Extensive laboratory and radiologic investigations failed to identify an underlying cause. Treatment with methylprednisolone, followed by azathioprine, was highly effective, resulting in complete resolution of radiographic abnormalities and sustained long-term remission.

## CASE DESCRIPTION

**Figure 1.**
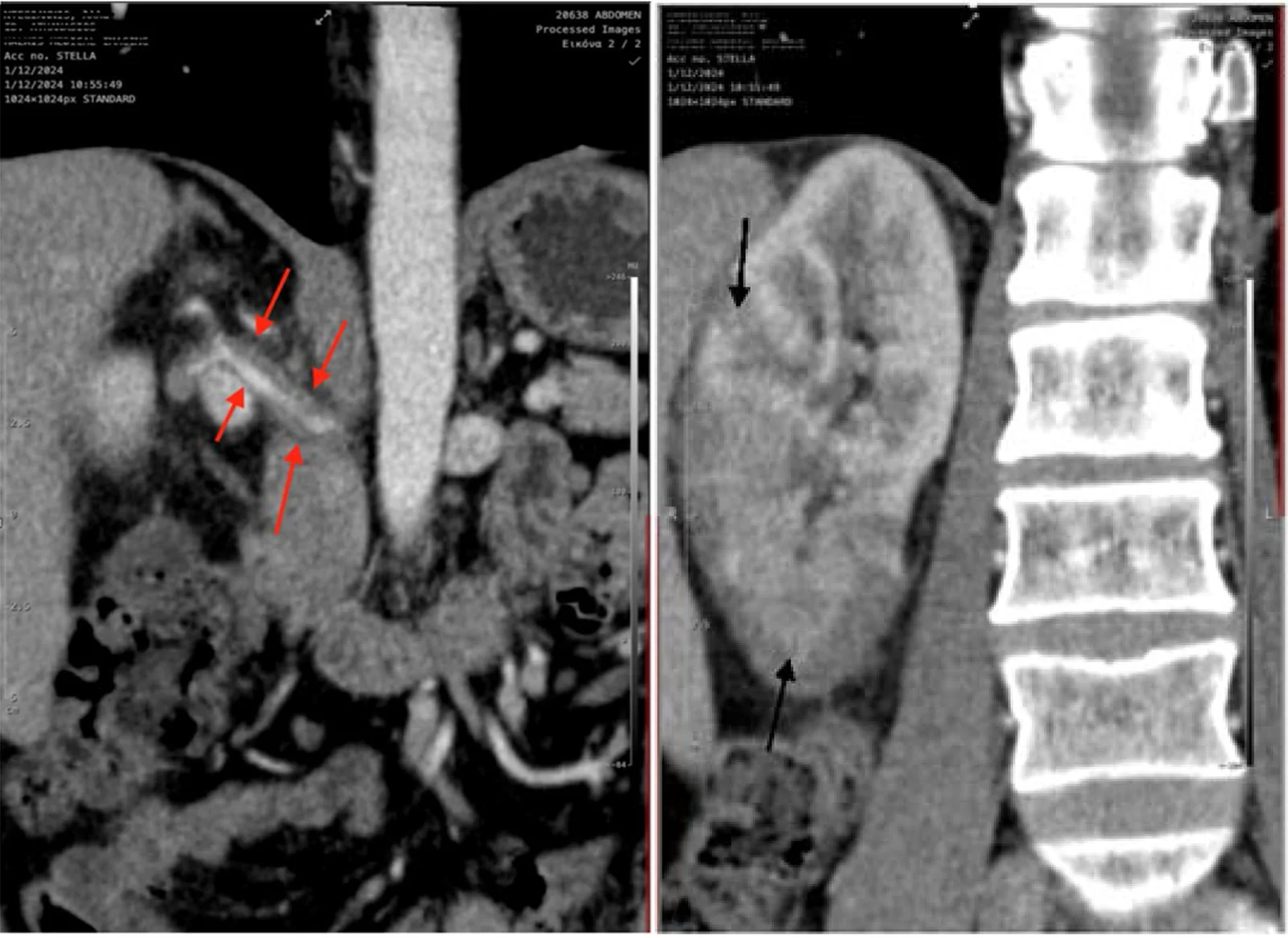
CT scan of the retroperitoneum showing right renal artery vasculitis with vessel wall thickening (red arrows) and multiple concomitant renal parenchymal infarcts (black arrows).

A 53-year-old Caucasian male presented to the emergency department with a history of right flank pain extending to the anterior abdominal wall for 2 days. He had no known comorbidities and reported no prescription or illegal drug use. Vital signs were within normal limits; blood pressure was equal in both arms, and the remainder of the physical examination was unremarkable. Laboratory evaluation, however, revealed leucocytosis (WBC count 19,000/μL), elevated inflammatory markers (erythrocyte sedimentation rate 49 mm/h, C-reactive protein 99 mg/L) and microscopic haematuria (5–6 red blood cells per high-power field). A computed tomography (CT) scan of the retroperitoneum demonstrated thickening of the right renal artery wall, highly suggestive of inflammatory vasculitis, with associated renal parenchymal hypoattenuation consistent with multiple infarctions (**Figure 1**). The patient was admitted to the Rheumatology department, and despite extensive laboratory investigations, no underlying cause was identified. The possibility of an underlying infection was excluded by multiple negative blood cultures; serologic testing for hepatitis B and C, syphilis, and QuantiFERON-TB was also negative. Autoimmune diseases as a secondary cause of vasculitis were excluded based on a negative immunologic profile (ANA, ENA, cANCA, pANCA, PR3 and MPO) and an unremarkable relevant history. Magnetic resonance angiography (MRA) of the chest and abdomen demonstrated normal appearances of the thoracic vessels, abdominal aorta and its branches, splenoportal axis, and left renal artery; the only pathological finding was wall thickening of the right renal artery. Vasculitis mimics, including segmental arterial mediolysis (SAM) and fibromuscular dysplasia (FMD) of the renal artery, were considered but deemed unlikely based on the radiologic findings and the presence of elevated inflammatory markers.

In light of the negative laboratory evaluation for an underlying infection and the presence of ischemic lesions in the right kidney, the patient was started on high-dose intravenous methylprednisolone, resulting in rapid relief of right flank pain and a prompt decline in inflammatory markers. Upon discharge, a gradual tapering of corticosteroids was initiated, and azathioprine was selected as a steroid-sparing agent. Six months later, the patient had discontinued corticosteroids while azathioprine was continued. At one year of follow-up, the patient had not experienced any clinical or laboratory relapse and repeat MRA was performed to assess for persistent disease activity or residual vascular damage. The MRA demonstrated complete resolution of the inflammatory process, with normal vessel integrity and no aneurysm formation. The patient continues to be monitored on a regular basis in the outpatient clinic, with no evidence of relapse.

## DISCUSSION

In this report, we present a rare case of isolated renal artery vasculitis in a previously healthy adult. The disease manifested as right flank pain radiating to the anterior abdominal wall, a common presenting symptom among patients seeking medical care in emergency departments. Such pain may result from a wide range of conditions, including urinary tract disorders, gastrointestinal causes, musculoskeletal or neurologic conditions, infections (varicella-zoster virus prior to rash onset), and pulmonary causes, among others.

In our case, physical examination was unremarkable; however, laboratory evaluation revealed leucocytosis (WBC count 19,000/μL), elevated inflammatory markers (erythrocyte sedimentation rate 49 mm/h, C-reactive protein 99 mg/L), and microscopic haematuria (5–6 red blood cells per high-power field), findings suggestive of an inflammatory renal process. Computed tomography of the retroperitoneum demonstrated thickening of the right renal artery wall, highly suggestive of inflammatory vasculitis. Extensive radiologic evaluation confirmed that the right renal artery was the only vessel affected.

An underlying infectious cause was excluded by multiple negative blood cultures and negative serologic testing for hepatitis B, hepatitis C, syphilis, and tuberculosis. Secondary vasculitis due to an autoimmune or neoplastic process was considered unlikely based on negative laboratory findings and the absence of radiologic evidence of an underlying disease. Vasculitis mimics of the renal artery, particularly segmental arterial mediolysis (SAM) and fibromuscular dysplasia (FMD), were also considered in the differential diagnosis but were deemed unlikely based on the laboratory and radiologic findings. SAM, an increasingly recognised noninflammatory arteriopathy of the splanchnic arteries, may present with similar symptoms, including acute flank pain and haematuria, due to arterial dissection caused by vacuolisation and lysis of the outer arterial media.^7^ In our case, however, the presence of elevated inflammatory markers, the radiologic findings, and the rapid and complete resolution of vessel wall thickening following immunosuppressive therapy argued against this diagnosis. Similarly, FMD, an idiopathic, noninflammatory vascular disease that commonly involves the renal arteries, may rarely present with flank pain and haematuria; however, the absence of the characteristic “string-of-beads” appearance on CT imaging and the presence of elevated inflammatory markers made this diagnosis unlikely.^8^

Given the rarity of this condition, we reviewed the current literature and identified only eight cases of unilateral renal artery inflammation without involvement of other vessels. These cases were attributed to two cases of Takayasu arteritis^9,10^, two of giant cell arteritis^11,12^, two associated with tuberculosis^13,14^, one due to ANCA-associated vasculitis^15^ and one following SARSCoV-2 vaccination^16^. All of these reports emphasised the unusual occurrence of single-vessel involvement and highlighted that prompt immunosuppressive therapy can prevent irreversible organ damage.

The significance of this report lies in the rarity of unilateral renal artery vasculitis without an identifiable underlying cause. In contrast, the eight cases identified in the literature occurred in the context of an underlying disease, including Takayasu arteritis, giant cell arteritis, ANCA-associated vasculitis, tuberculosis, or following SARS-CoV-2 vaccination. Our case differs fundamentally from these reports, as it represents a truly isolated, idiopathic, unifocal vasculitis confined to a single renal artery.

**Figure 2.**
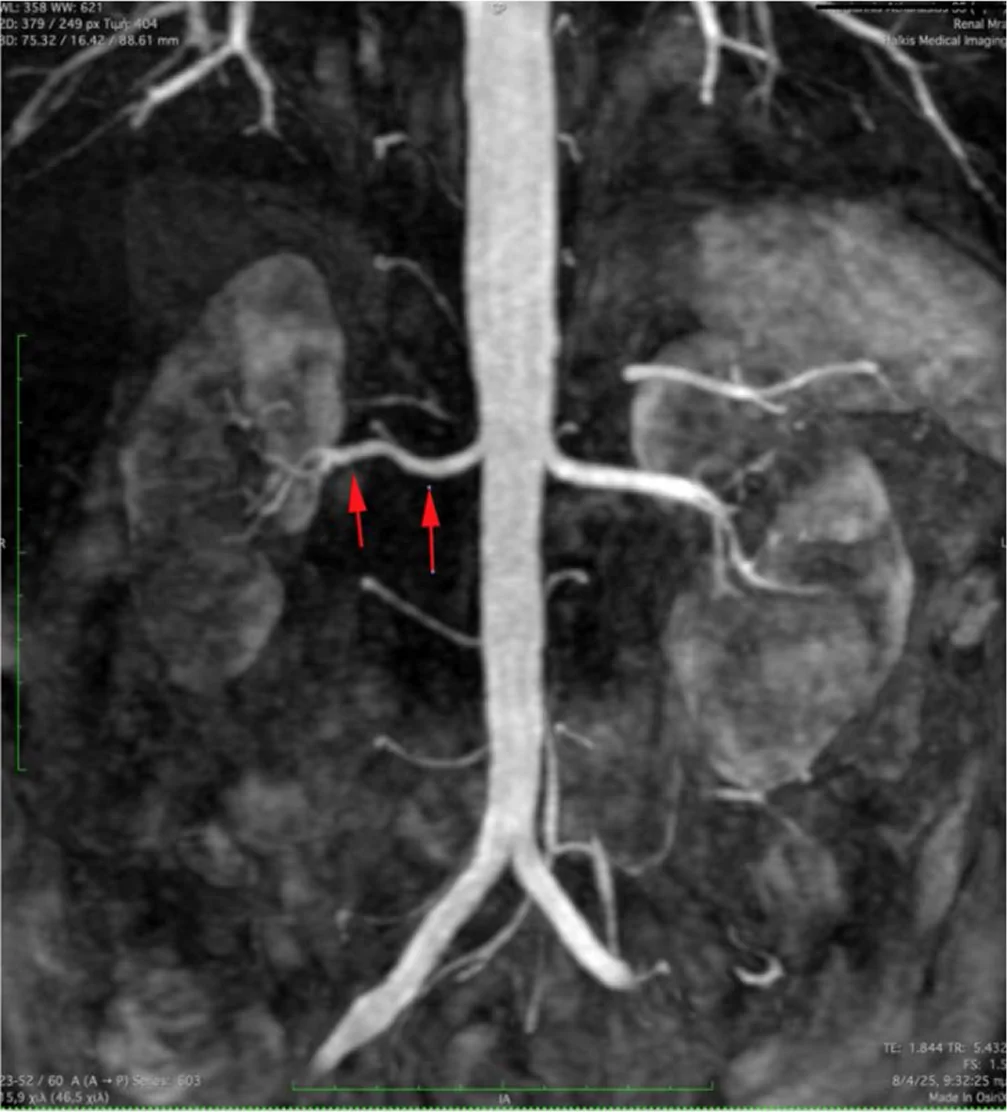
Magnetic resonance angiography (MRA) of the renal arteries, performed one year after the initial inflammatory event, demonstrating complete resolution of the inflammatory process with normal-appearing vessel walls and patent lumen (red arrows).

Management of such a rare and unclassified form of vasculitis is challenging. Following the initiation of glucocorticoid therapy and rapid symptom relief, azathioprine was added as a steroid-sparing agent based on its established efficacy as maintenance therapy in primary vasculitic syndromes.^17^ Gradual tapering of glucocorticoids over six months was well tolerated, with no clinical or laboratory evidence of disease relapse. At one year of follow-up, while the patient remained in complete remission on azathioprine, repeat MRA of the affected area was performed to assess for persistent disease activity or anatomic vascular compromise; imaging demonstrated complete resolution of the inflammatory process, with normal vessel walls and a patent lumen, and no residual damage (**Figure 2**).

## CONCLUSION

Isolated renal artery vasculitis is an exceedingly rare entity that may present with non-specific symptoms, such as flank pain and microscopic haematuria, without specific laboratory abnormalities. Early recognition and prompt immunosuppressive therapy can prevent renal damage and lead to complete clinical and radiologic remission. Our report contributes to the limited literature on this rare entity and reinforces the need for continued awareness among clinicians of atypical presentations of vasculitis.
